# Supplementary material for: MXene based saturation organic vertical photoelectric transistors with low subthreshold swing
Source: Nat Commun. 2022 May 24;13:2898. doi: 10.1038/s41467-022-30527-w (PMC9130145; doi:10.1038/s41467-022-30527-w)
Supplement: Supplementary file 1 — Supplementary information [file 41467_2022_30527_MOESM1_ESM.pdf]

Supplementary Information

MXene based Saturation Organic Vertical Photoelectric Transistors with Low Subthreshold Swing

Enlong Li[1,2], Changsong Gao[1,2], Rengjian Yu[1,2], Xiumei Wang[1,2], Lihua He[1,2], Yuanyuan Hu[3], Huajie Chen[4], Huipeng Chen[1,2*], Tailiang Guo[1,2]

[1]Institute of Optoelectronic Display,National & Local United Engineering Lab of Flat Panel Display Technology, Fuzhou University, Fuzhou 350002, China

[2] Fujian Science & Technology Innovation Laboratory for Optoelectronic Information of China, Fuzhou 350100, China

[3]College of Semiconductors (College of Integrated Circuits), Hunan University, Changsha 410082, China

[4]College of Chemistry, Xiangtan University, Xiangtan 411105, China

Email: hpchen@fzu.edu.cn

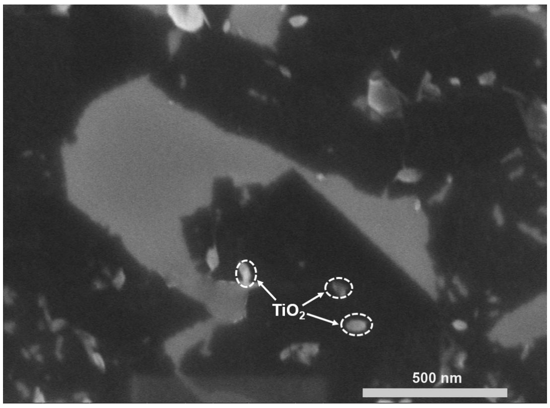

Supplementary Fig. 1. The high resolution SEM image of MXene with a concentration of 3 mg/ml. The small white particles are the partially oxidized MXene. The grey part in the middle of picture is SiO_2 substrate, which is the perforation area of MXene.

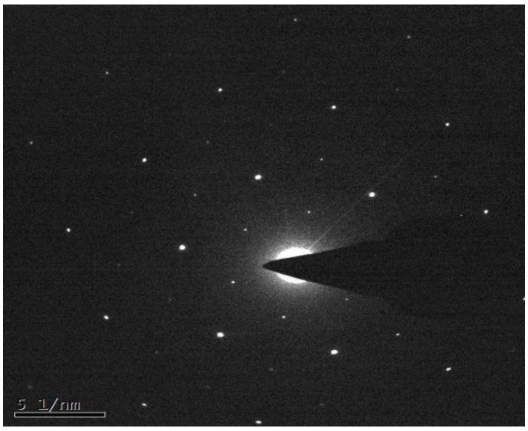

Supplementary Fig. 2. The selective area electron diffraction (SAED) pattern of MXene.

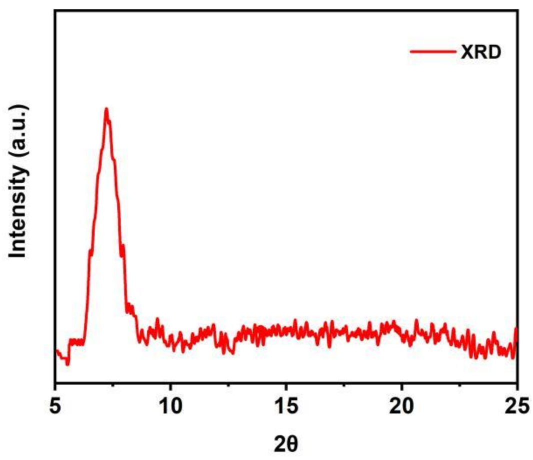

Supplementary Fig. 3. The XRD spectrum of MXene film, exhibiting a characteristic peak (002) of $Ti_3C_2T_x$ at 2θ = 7.1

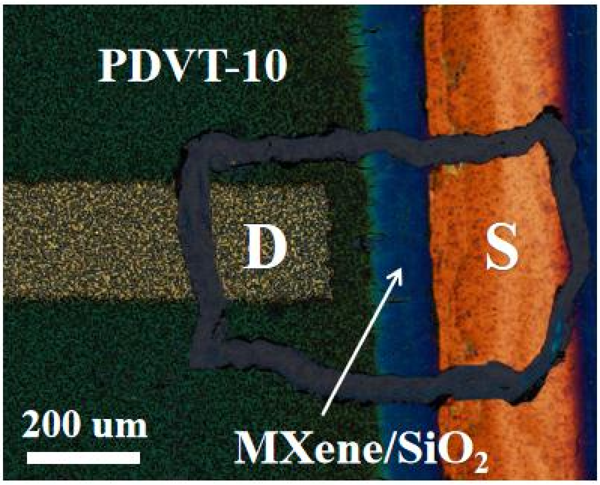

Supplementary Fig. 4. The microscope image of MVOFET.

As shown in the microscope image, the distance between source and drain electrode is about 200 um. To calculate the current density, the area of drain electrode is independent as about 200×200 um, the thickness of PDVT-10 deposited on MXene and AgNWs is 76 nm and 125 nm, respectively, the difference of semiconductor thickness is ascribed to the hydrophilic nature of MXene and the plasma treatment of substrate before the spin coating of MXene.

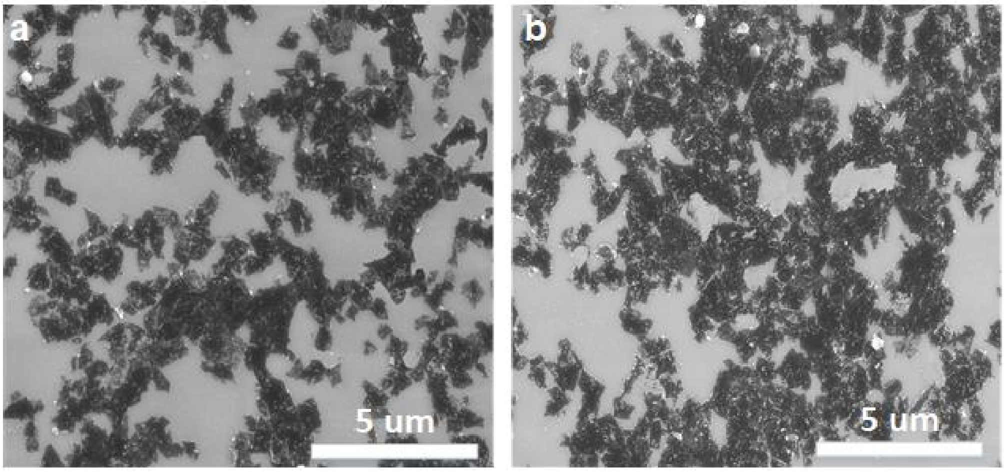

Supplementary Fig. 5. The SEM images of MXene with different concentration.(a) The SEM images of MXene under1 mg/ml. (b) The SEM images of MXene under 2 mg/ml.

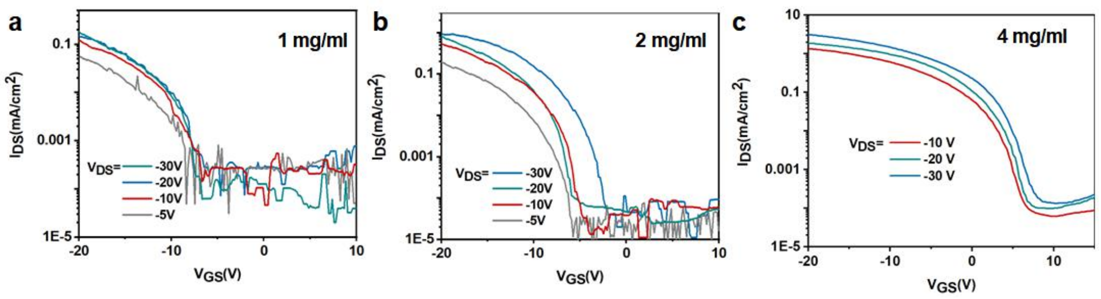

Supplementary Fig. 6. **Devices performance of MVOFET with different MXene concentration**. The transfer curves of MVOFET under different V_{DS} with a concentration of (a) 1mg/ml, (b) 2 mg/ml and (c) 4 mg/ml MXene, respectively.

A higher concentration resulted in a more continuous and thicker MXene film, which reduced the gate control ability as the shield effect of gate electric field was enhanced. On the other hand, the thickness and roughness of MXene film will increase with more stacked MXene flake, leading to higher off current. Therefore, the performance of device decreased when the concentration was too higher

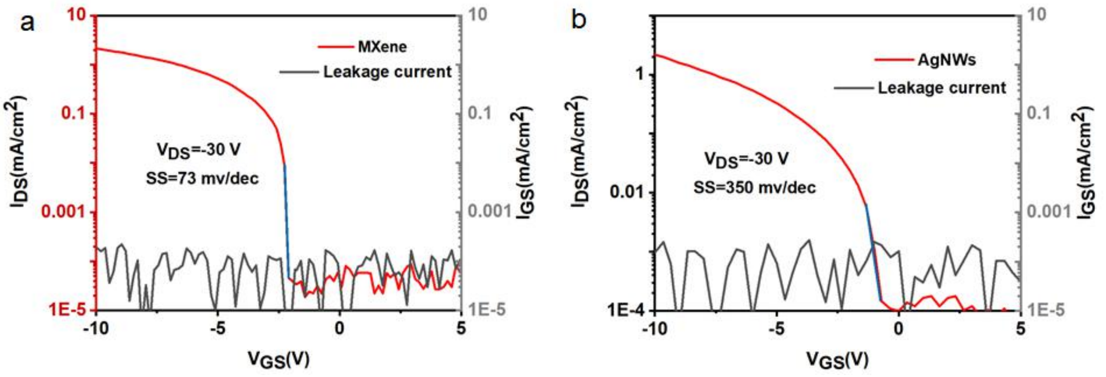

Supplementary Fig. 7. **SS and leakage current of MVOFET and AgNWs based device.** (a-b) The SS and leakage current of VOFET made by MXene and AgNWs, respectively.

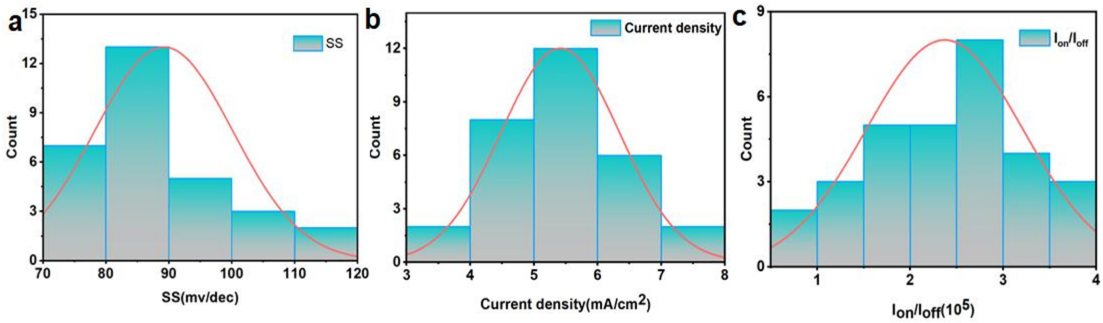

Supplementary Fig. 8. **The distribution histogram of basic transistor performance under 30 MVOFET devices.** The distribution of (a) SS, (b) current density and (c) I_{on}/I_{off} of MVOFET.

Due to the inhomogeneity of the MXene solution and the randomness of the MXene distribution during spin coating, different devices have certain performance differences. According to the distribution histogram of 30 MVOFET devices, the SS was mostly distributed between 70-90 mv/dec, which was still much smaller than AgNWs based VOFET. The distribution of current density and Ion/Ioff ratio were also counted which exhibited acceptable device variation.

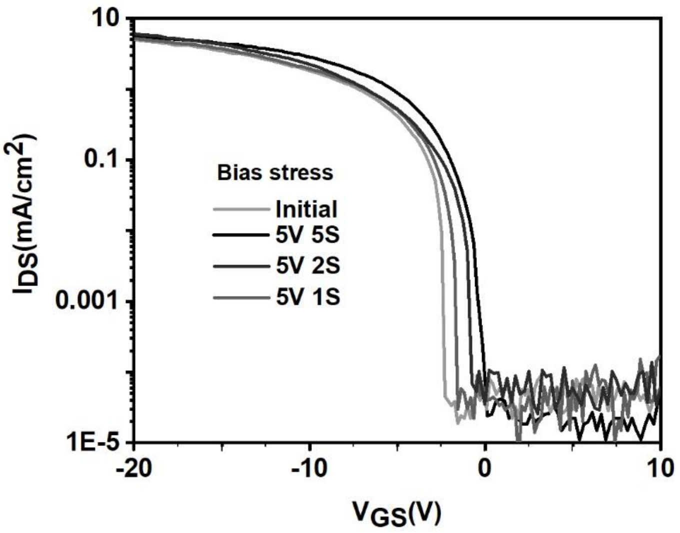

Supplementary Fig. 9. **The bias stability of MVOFET measured at 5V gate voltage with different bias time**. The threshold voltage exhibits a small right shift under positive gate voltage, this can be ascribed to the accumulation of carriers in the perforation of MXene film.

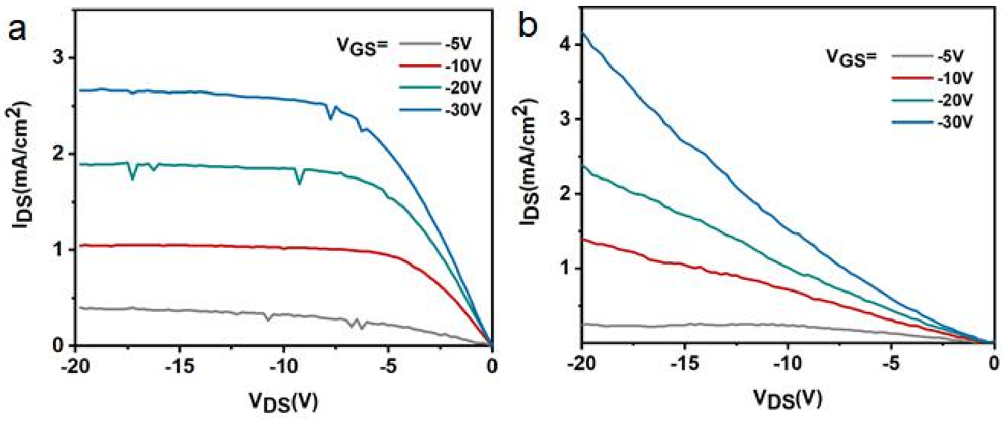

Supplementary Fig. 10. **Output characteristics of VOFET** (a) and (b) the output current of MVOFET and AgNWs based VOFET, respectively.

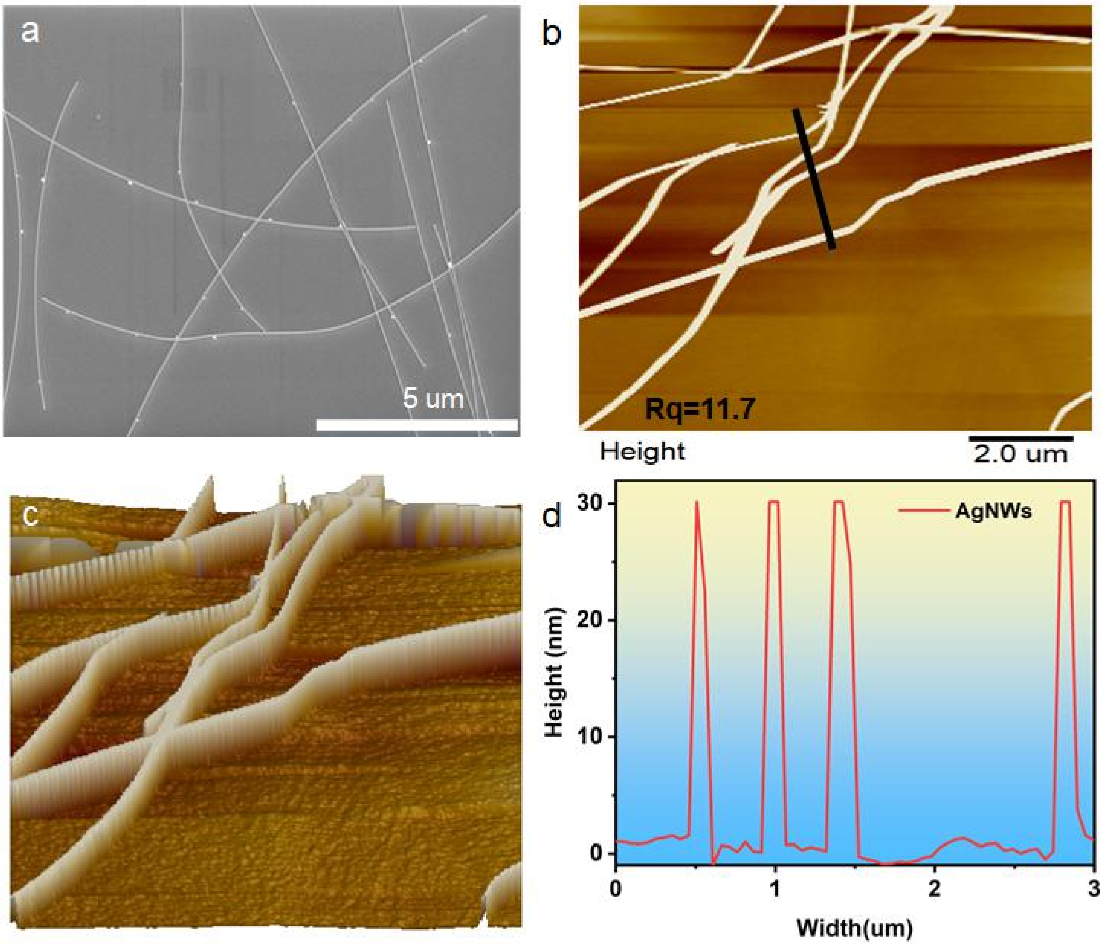

Supplementary Fig. 11. **The morphology of AgNWs.** (a) SEM image of AgNWs with a concentration of 0.5 mg/ml. (b) AFM image of AgNWs. (c) A 3D image of side view of the AFM of AgNWs. (d) The height profile measured by the black solid line in Supplementary Fig. 11b.

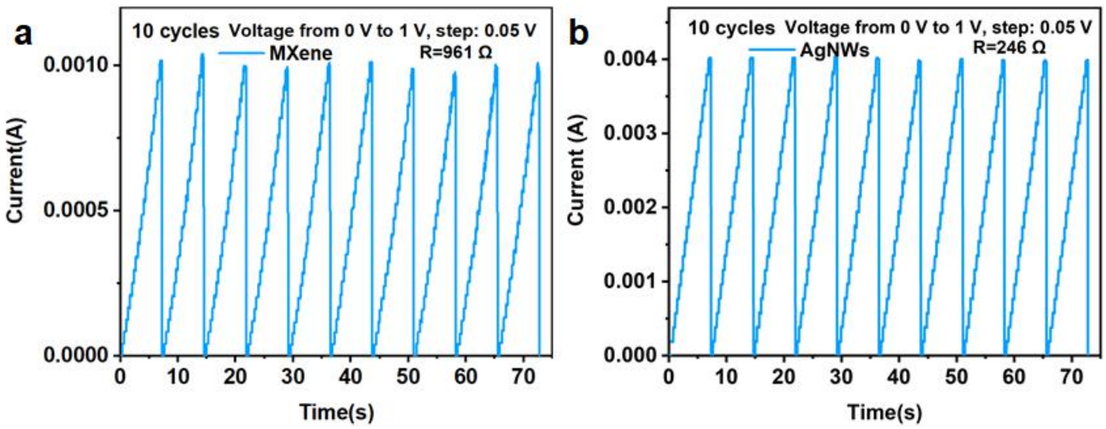

Supplementary Fig. 12 The conductivity of (a) MXene and (b) AgNWs.

Table S1. The comparison of basic transistor performance between MVOFET and other vertical organic transistor.

Semiconductor Materials	Source electrode	SS	Ion/Ioff	Current density	Threshold voltage(V)	Ref
C_{60}	Grahphene	>2(V/dec)[a]	3×10^3	1×10^{-6}（A）	>50[a]	1
PBDB-T	Grahphene	>2(V/dec)[a]	10^4	1×10^{-6}（A）	4	2
$PC_{71}BM$	Grahphene	>2(V/dec)[a]	10^5	1×10^{-5}（A）	-5	2
C_{60}	Grahphene	5.75(V/dec)	8×10^4	3×10^{-6}（A）	-10	3
PTCDI-C_8	Grahphene	>2(V/dec)[a]	$<10^3$	12.4 (mA/cm2)	>-20[a]	4
Pentacene	Grahphene	>2(V/dec)[a]	$<10^3$	10.5 (mA/cm2)	>30[a]	4
DNTT	CNT	500(mV/dec)	10^5	110 (mA/cm2)	1	5
MoS_2	CNT	>2(V/dec)[a]	10^3	1×10^{-6}（A）	>-40[a]	6
$MAPbI_3$	Porous ITO	1.1(V/dec)	10^4	1 (mA/cm2)	1	7
CuPc	Pattern Au	>2(V/dec)[a]	10	0.5 (A/cm2)	1	8
PDVT-8	AgNWs	500(mV/dec)	2×10^4	6.5 (mA/cm2)	1.5	9
PDVT-10	Graphene	1.75(V/dec)	1×10^4	37.5(mA/cm2)	8.5	This work
PDVT-10	AgNWs	350(mV/dec)	1×10^5	9.2 (mA/cm2)	0.7	This work
PDVT-10	MXene	73 (mV/dec)	2×10^5	5.8 (mA/cm2)	1.2	This work

[a] estimated from the transfer curves of reported devices.

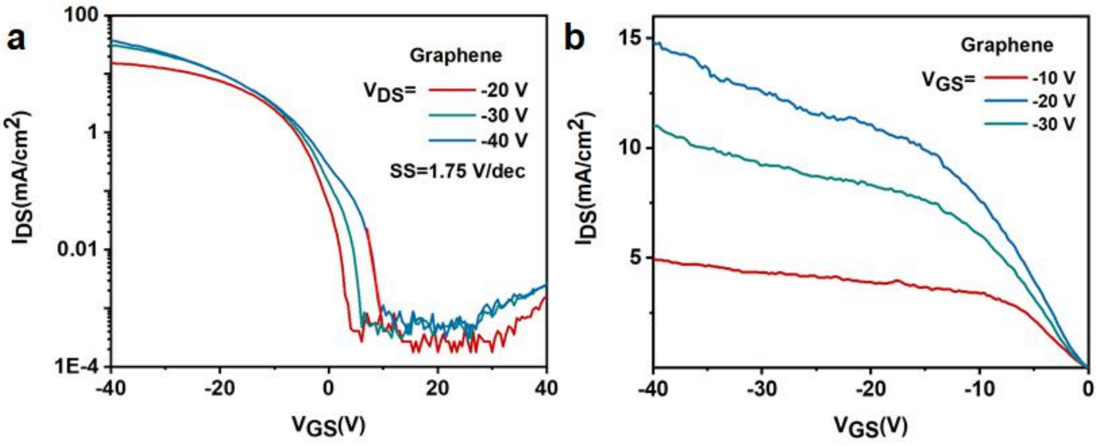

Supplementary Fig. 13 The device performance of graphene based VOFET. (a) and (b)The transfer curves and output current of graphene based VOFET.

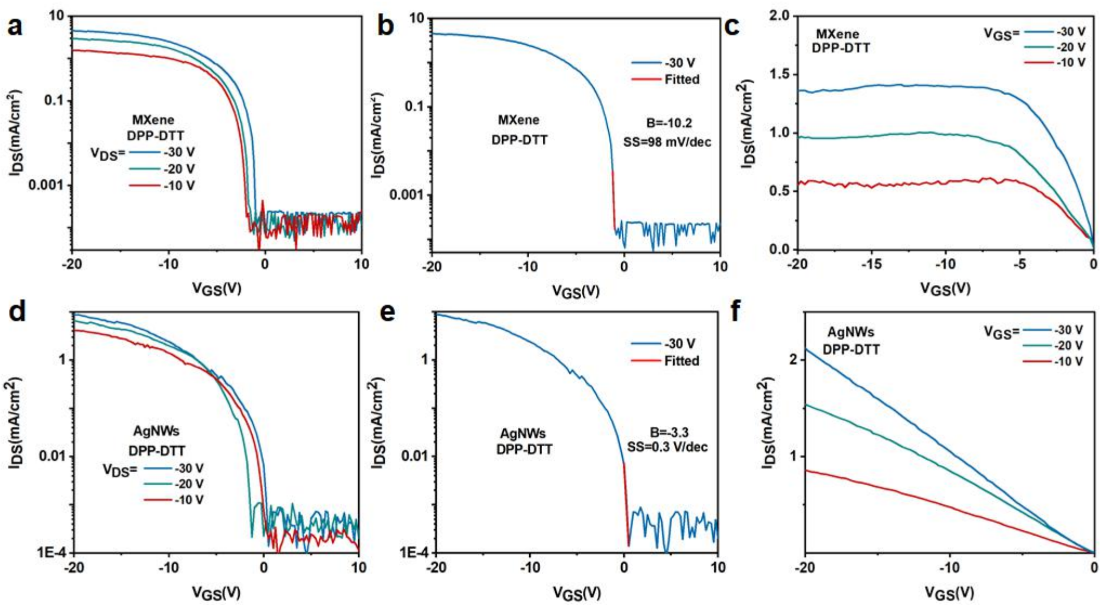

Supplementary Fig. 14. **The device performance of DPP-DTT based VOFET with MXene and AgNWs as source electrode**. (a) The transfer curves of VOFET under different V_{DS}. (b) The transfer curve at V_{DS}=-30 V, exhibiting an on/off ratio about 5×10^4, small SS of 98 mv/dec. (c) Output characteristic of VOFET exhibiting a significant saturation current under small V_{DS}. (d) The transfer curves of VOFET utilizing AgNWs as source electrode. (e) The transfer curve of VOFET made by AgNWs measured at V_{DS}=-30 V. (f) Output characteristic of AgNWs based VOFET under different V_{GS}.

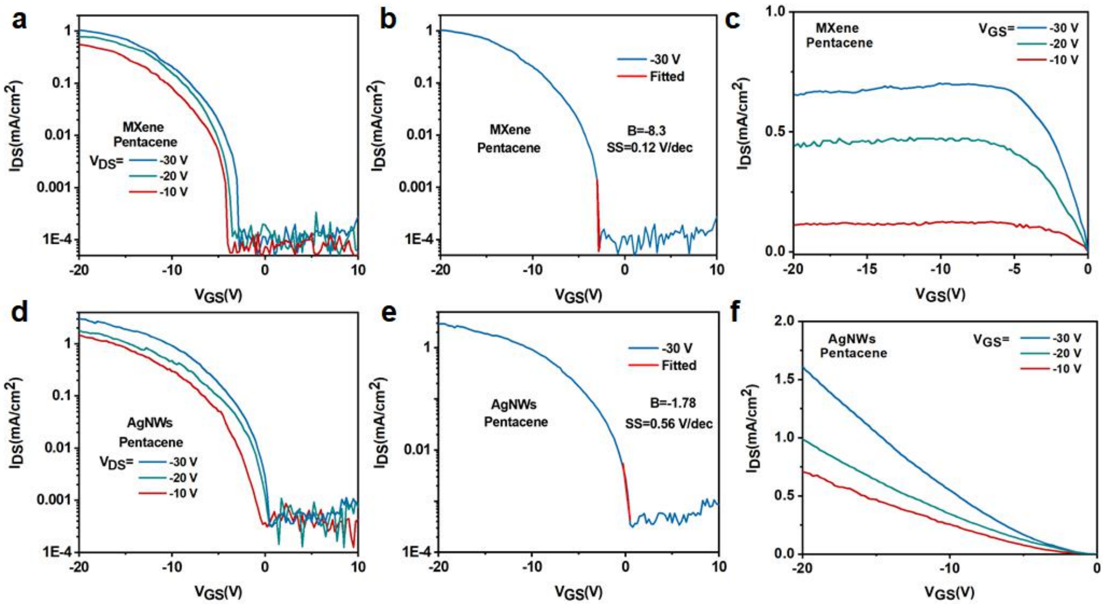

Supplementary Fig. 15. **The device performance of pentacene based VOFET with MXene and AgNWs as source electrode**. (a) The transfer curves of VOFET under different V_{DS}. (b) The transfer curve at V_{DS}=-30 V, exhibiting an on/off ratio about 10^4 and small SS of 0.12 V/dec. (c) Output characteristic of VOFET exhibiting a significant saturation current under small V_{DS}. (d) The transfer curves of VOFET utilizing AgNWs as source electrode. (e) The transfer curve of VOFET made by AgNWs measured at V_{DS}=-30 V. (f) Output characteristic of AgNWs based VOFET under different V_{GS}.

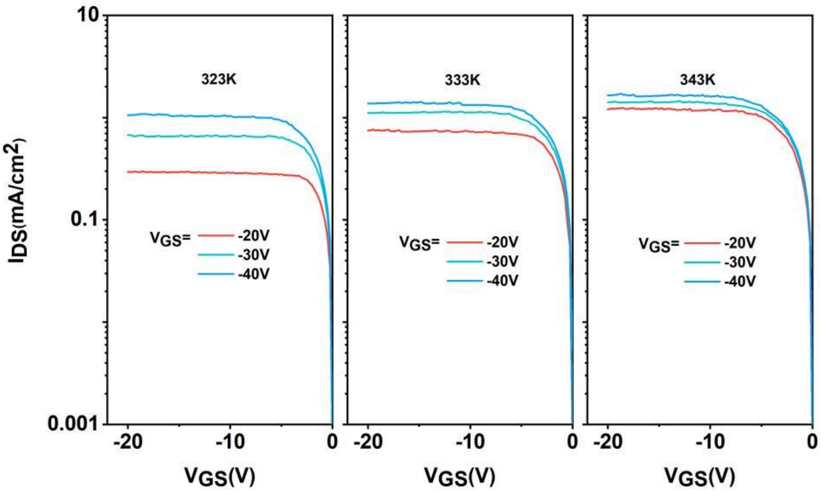

Supplementary Fig. 16. The output characteristics of MVOFET measured at different temperatures.

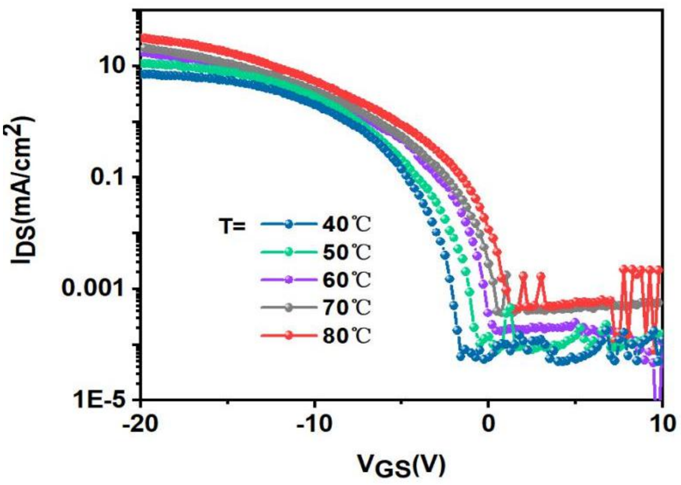

Supplementary Fig. 17. The transfer curves of MVOFET as a function of different temperature varied from 313K to 353K.

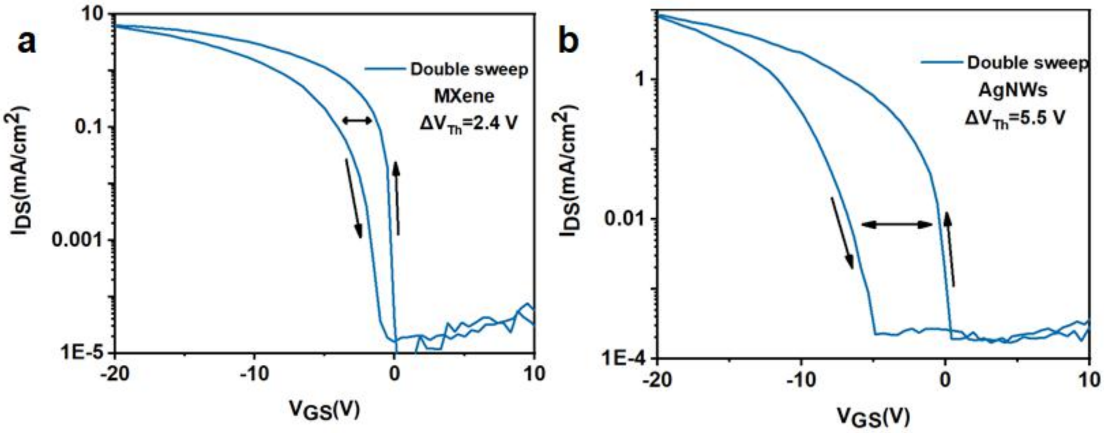

Supplementary Fig. 18. Double sweep measurement. (a) the double sweep measurement of MXene and (b) AgNWs. The variation of threshold voltage of MVOFET is two times smaller than AgNWs based VOFET.

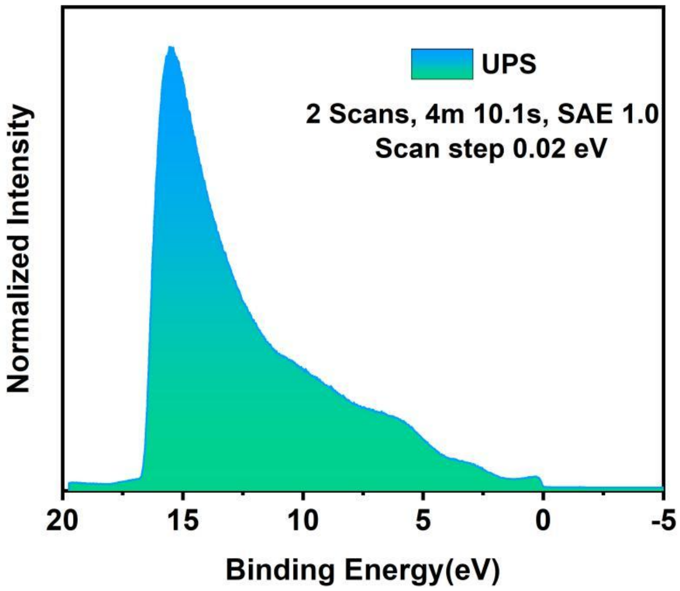

Supplementary Fig. 19. UPS of MXene film.

UPS measurements were performed to determine the working function of MXene film, and the working function can be obtained by the following formula:

$$W = hv - E_{cutoff} + E_{Fermi} \quad (1)$$

in which hv is the ultraviolet radiation energy (21.22 eV), E_{cutoff} is the binding energy of the secondary in the spectra, and E_{Fermi} is the difference between the valance band and the Fermi level.

Theoretical modeling in COMSOL simulation

The finite-element simulations are performed in COMSOL Multiphysics. A 2-dimensional space coupled to the quasi-statics electric mode is employed to simulate the currents and potential distribution in the AgNWs and MXene based vertical organic transistors. The calculations are demonstrated along a representative device cross-section composed of gate, gate dielectric, source, source insulator layer, semiconductor and drain from the bottom to the top of the vertical organic transistor, respectively. The component materials are defined similarly to the fabricated devices. The Si and SiO_2 are used to compose the gate electrode and dielectric layers, respectively. AgNWs and MXene are used as the source electrode, TiO_2 is used as the source electrode covered insulator layer, a P-type material CuPc is used as the semiconductor layer, and Au is used as material for drain electrodes. The electrical properties, such as conductivity (σ) and relative permittivity (ε) of Si, SiO_2 and Au are given by the software library. For the source electrode of AgNWs and MXene, the σ are set to be 1.9×10^5 S/m and 2.4×10^5 S/m and the ε are 4 and 3.5, respectively. The σ and ε of TiO_2 are set to be 1×10^{-11} S/m and 20. As for semiconductor layer, we have used $\sigma=10^{-3}$ S/m and $\varepsilon= 4.5$. The interfacial resistance of the source and drain electrodes are adjusted to recover the normalize current distribution of vertical organic transistor. To complete the simulation, gate and drain voltages are set to be $V_{GS} = V_{DS} = -10$ V, with the source electrode being grounded.

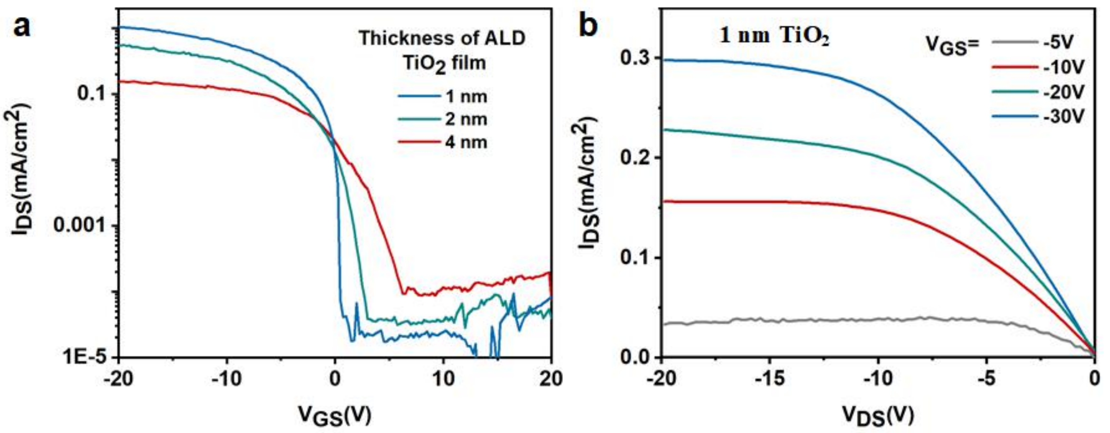

Supplementary Fig. 20. **The device performance of MVOFET with different TiO_2 thickness.** (a) The transfer curves of MVOFET with different thickness of TiO_2 (b) The output current of MVOFET with 1 nm TiO_2.

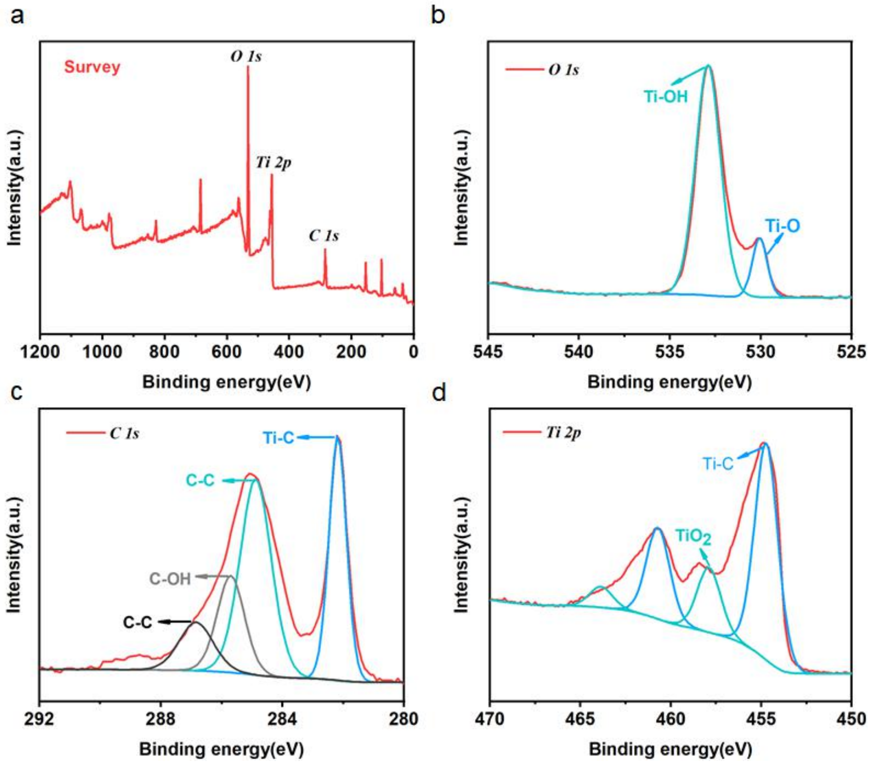

Supplementary Fig. 21. **XPS analysis of MXene**. (a) Survey XPS spectra of Ti3C2Tx MXene (b-d) The focused spectra of *O 1s*, *C 1s* and *Ti 2p*, respectively.

Supplementary Fig. 21a is the survey spectra of MXene where the main peaks at 531.5, 457.6 and 283.7 eV are ascribed to the *O 1s, Ti 2p* and *C 1s*, respectively. As shown in Supplementary Fig. 21b, the *O 1s* XPS spectrum of MXene exhibited two peaks at 530.1 eV and, 532.5 eV corresponding to Ti-O and Ti-OH, respectively, confirming the presence of –OH group on the surface. The *C 1s* spectrum of $Ti_3C_2T_x$ were fitted with four components centered at 282.2 eV, 284.9 eV, 285.7 eV and 286.9 corresponding to Ti-C, C-C, C-OH and C-O, respectively (Supplementary Fig. 21c). More details, as shown in Supplementary Fig. 21d, the focused spectra of *Ti 2p* is illustrated to clarify the chemical bonds of MXene. The *Ti 2p* spectra were fitted with three doublets (*Ti* $2p_{3/2}$−*Ti* $2p_{1/2}$) with a fixed area ratio 2:1. The peak at 458.5 eV and 454.7 eV can be assigned as Ti ions in valence 4^+ (TiO_2) and Ti-C bond[10].

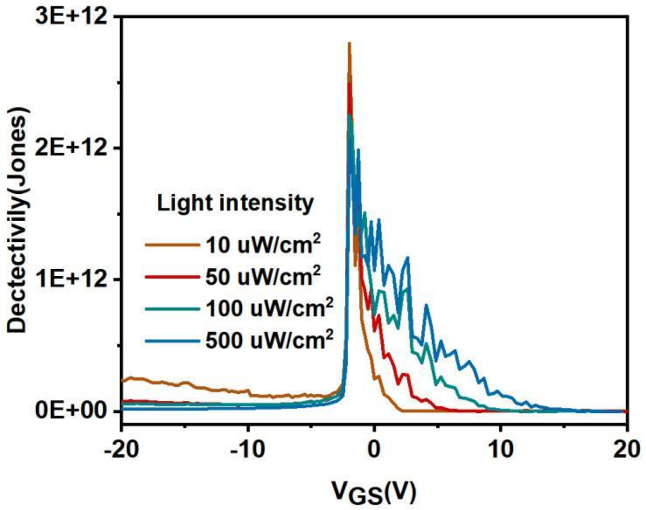

Supplementary Fig. 22. The detectivity of MVOFET as a function of light intensity.

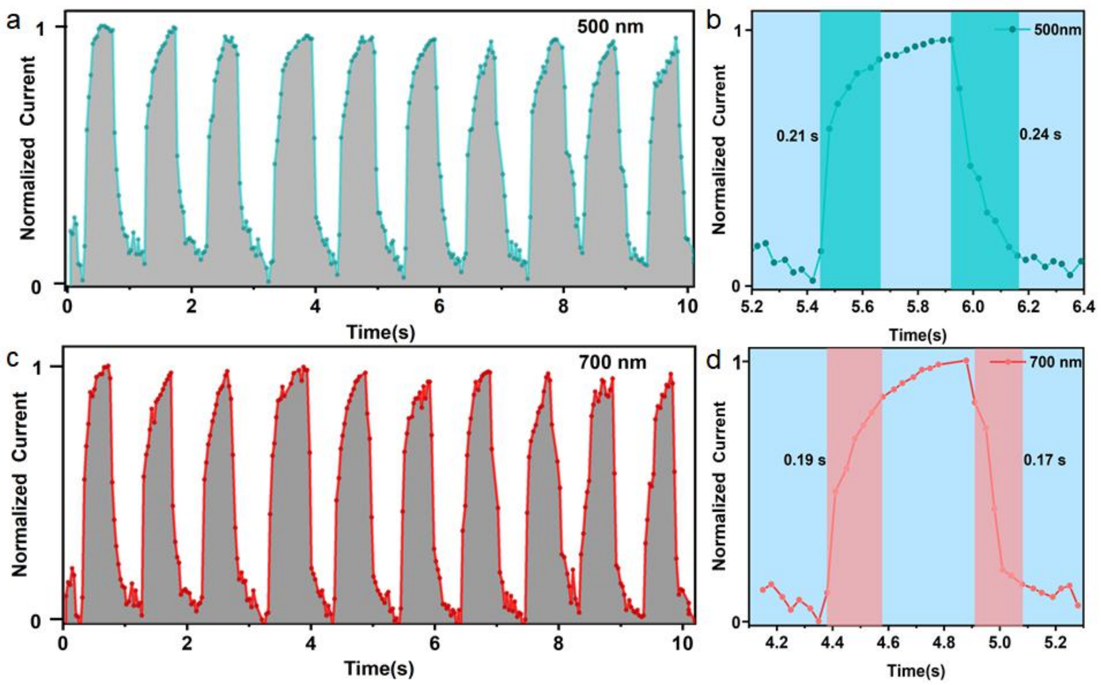

Supplementary Fig. 23. **The current-time response under visible light**. (a and c) The real-time light response cycle test measured at V_{GS}=10 V and V_{DS}=-20 V under 500 nm and 700 nm, respectively. (b and d) A single light response under 500 nm and 700 nm, respectively.

Supplementary Fig. 23a shows the real-time photoresponse under 500 nm and 10 test cycles. Unlike under UV light, where the current rise and fall instantaneously when light is turned on and off, the variation of current is relative slow at 500 nm. The switch-on and switch-off time under 500 nm is extracted to be 0.21 s and 0.24 s (Supplementary Fig. 23b), which is 20 times slower than that of UV light while it is still much faster than most planar field effect phototransistors due to the ultra-short channel length of MVOFET. Similarly, the response times under 700 nm are 0.19 s and 0.17 s, which is relative faster than that of 500 nm as a result of the strong absorption at 700 nm (Supplementary Fig. 23c-d).

Table S2. The comparison between MVOFET and other reported phototransistors.

Structure	Materials	R (A/W)	D (Jones)	I_{light}/ I_{dark}	λ(nm)	Speed (s)	Ref
Planner	DPP-DTT/$CsPbI_3$	110	2.9×10^{13}	6×10^{3}	350–940	3.2	[11]
Planner	C60	8×10^{-3}	N/A	10	350-650	1.8	[12]
Planner	Graphene oxide	1×10^{-2}	N/A	N/A	400-1600	0.018	[13]
Planner	SnS_2	860	1.1×10^{10}	<10	300-750	0.7	[14]
Planner	2F-4-TFPTA Single crystal	3.6×10^{3}	N/A	10	465-622	0.043	[15]
Planner	AZO/ ZnO NP	8×10^{-2}	N/A	80	365	0.11	[16]
Vertical	$CsPbBr_3$ NCs /Graphene	2.2×10^{3}	1.1×10^{9}	N/A	405	0.02	[17]
Vertical	PbSe QDs /AgNWs	28	1.3×10^{13}	10^{4}	1064	0.11	[18]
Vertical	DPA single crystal /Graphene	110	1×10^{13}	10^{4}	420	>1	[19]
Vertical	PDVT-8/AgNWs	150	1.3×10^{11}	2×10^{3}	400-700	2	[9]
Vertical	PTCDI-C_8 and Pentence/Graphene	0.4	1.2×10^{9}	10^{4}	480-670	0.1	[20]
Vertical	PDVT-10/MXene	366	2.8×10^{12}	7×10^{4}	365-700	0.01	This work

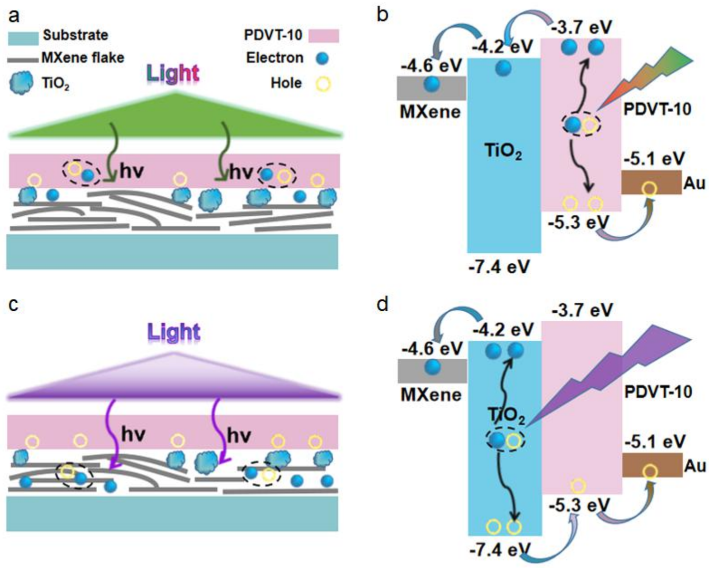

Supplementary Fig. 24. **Photodetection mechanism of MVOFET.** (a and c) The mechanism schematic of the photoresponse of MVOFET under under visible light and UV, respectively. (b and d) The energy level and carrier transport under visible and UV light, respectively.

As shown in Supplementary Fig. 24a, when the device is exposed to visible light, photo induced electron-hole pairs are generated in the PDVT-10 layer. The electron-hole pairs are further separated under the variation of energy level and a bias of 10 V gate voltage, as a result the electrons are transferred to TiO_2 and further to the MXene source electrode, while the holes migrate to opposite direction to top Au drain electrode, resulting in the generation of photocurrent (Supplementary Fig. 24b). Moreover, with UV irradiation, the photogenerated carriers are induced in TiO_2 composites (Supplementary Fig. 24c**)**. The generated photocarriers in TiO_2 are separated to source and drain electrode benefiting from the large intrinsic built-in field across the TiO_2 and PDVT-10 heterojunction interface and the gate bias (Supplementary Fig. 24d**).**

Supplementary references

1. Kim K*, et al.* Structural and electrical investigation of c60–graphene vertical heterostructures. *ACS Nano* **9**, 5922-5928 (2015).
2. Lim DU, Kim S, Choi YJ, Jo SB, Cho JH. Percolation-limited dual charge transport in vertical p-n heterojunction schottky barrier transistors. *Nano Lett.* **20**, 3585-3592 (2020).
3. Parui S*, et al.* Gate-controlled energy barrier at a graphene/molecular semiconductor junction. *Adv. Funct. Mater.* **25**, 2972-2979 (2015).
4. Kim JS, Kim BJ, Choi YJ, Lee MH, Kang MS, Cho JH. An organic vertical field-effect transistor with underside-doped graphene electrodes. *Adv. Mater.* **28**, 4803-4810 (2016).
5. McCarthy MA, Liu B, Rinzler AG. High current, low voltage carbon nanotube enabled vertical organic field effect transistors. *Nano Lett.* **10**, 3467-3472 (2010).
6. Phan TL*, et al.* Efficient gate modulation in a screening-engineered mos2/single-walled carbon nanotube network heterojunction vertical field-effect transistor. *ACS Appl. Mater. & Interf.* **11**, 25516-25523 (2019).
7. Yu H, Cheng Y, Shin D, Tsang S-W, So F. Vertical organic-inorganic hybrid perovskite schottky junction transistors. *Adv. Electron. Mater.* **4**, 1800039 (2018).
8. Nawaz A, Merces L, de Andrade DM, de Camargo DHS, Bof Bufon CC. Edge-driven nanomembrane-based vertical organic transistors showing a multi-sensing capability. *Nat. Commun.* **11**, 841 (2020).
9. Fang Y*, et al.* Inkjet-printed vertical organic field-effect transistor arrays and their image sensors. *ACS Appl.Mmater. &Interf.* **10**, 30587-30595 (2018).
10. Jia Z*, et al.* Constructing conductive titanium carbide nanosheet (mxene) network on polyurethane/polyacrylonitrile fibre framework for flexible strain sensor. *J Colloid Interf. Sci.* **584**, 1-10 (2021).
11. Zou C*, et al.* A highly sensitive uv-vis-nir all-inorganic perovskite quantum dot phototransistor based on a layered heterojunction. *Adv. Opt. Mater.* **6**, 1801219 (2018).
12. Zheng S*, et al.* Solution-grown large-area c60 single-crystal arrays as organic photodetectors. *Carbon* **126**, 299-304 (2018).
13. Li Y*, et al.* A broadband phototransistor based on three-dimensional reduced graphene oxide foam. *Nanomater.* **8**, 913 (2018).
14. Yu J, Suleiman AA, Zheng Z, Zhou X, Zhai T. Giant - enhanced sns2 photodetectors with broadband response through oxygen plasma treatment. *Adv. Funct. Mater.* **30**, 2001650 (2020).
15. Kim JH, Oh S, Park SK, Park SY. Green - sensitive phototransistor based on solution - processed 2d n - type organic single crystal. *Adv. Electron. Mater.* **5**, 1900478 (2019).
16. Zhang X*, et al.* High-performance flexible ultraviolet photodetectors based on azo/zno/pvk/pedot:Pss heterostructures integrated on human hair. *ACS Appl.Mater. & Interf.* **11**, 24459-24467 (2019).
17. Che Y, Cao X, Zhang Y, Yao J. High performance cspbbr3 perovskite nanocrystal

vertical phototransistor with graphene electrode. *Opt. Mater.* **100**, 109664 (2020).

18. Subramanian A, *et al.* Solution-processed vertical field-effect transistor with separated charge generation and charge transport layers for high-performance near-infrared photodetection. *ACS Appl. Electron. Mater.* **2**, 3871-3879 (2020).
19. Liu J, *et al.* Organic-single-crystal vertical field-effect transistors and phototransistors. *Adv. Mater.* **30**, e1803655 (2018).
20. Kim JS, *et al.* Schottky-barrier-controllable graphene electrode to boost rectification in organic vertical p-n junction photodiodes. *Adv. Funct. Mater.* **27**, 1704475 (2017).
